# *Leukemogenic Ptpn11* Allele Causes Defective Erythropoiesis in Mice

**DOI:** 10.1371/journal.pone.0109682

**Published:** 2014-10-07

**Authors:** Tatiana Usenko, Gordon Chan, Emina Torlakovic, Ursula Klingmüller, Benjamin G. Neel

**Affiliations:** 1 Princess Margaret Cancer Center, University Health Network, Toronto, Canada; 2 Department of Laboratory Hematology, Toronto General Hospital, University Health Network, Toronto, Canada; 3 Systems Biology of Signal Transduction, German Cancer Research Center (DKFZ), DKFZ-ZMBH-Alliance, Heidelberg, Germany; 4 Departments of Medical Biophysics and Biochemistry, University of Toronto, Toronto, Canada; Institut national de la santé et de la recherche médicale (INSERM), France

## Abstract

Src homology 2 (SH2) domain-containing phosphatase 2 (SHP2), encoded by *PTPN11*, regulates signaling networks and cell fate in many tissues. Expression of oncogenic *PTPN11* in the hematopoietic compartment causes myeloproliferative neoplasm (MPN) in humans and mice. However, the stage-specific effect(s) of mutant *Ptpn11* on erythroid development have remained unknown. We found that expression of an activated, leukemogenic *Ptpn11* allele, *Ptpn11^D61Y^*, specifically in the erythroid lineage causes dyserythropoiesis in mice. *Ptpn11^D61Y^* progenitors produce excess cKIT^+^CD71^+^Ter119^−^ cells and aberrant numbers of cKIT^l^°CD71^+^ erythroblasts. Mutant erythroblasts show elevated activation of ERK, AKT and STAT3 in response to EPO stimulation, and MEK inhibitor treatment blocks *Ptpn11^D61Y^*-evoked erythroid hyperproliferation *in vitro.* Thus, the expression of oncogenic *Ptpn11* causes dyserythropoiesis in a cell-autonomous manner *in vivo.*

## Introduction

Src homology 2 (SH2) domain-containing phosphatase 2 (SHP2), encoded by the *PTPN11* gene, is a non-receptor protein-tyrosine phosphatase (PTP) that acts as a crucial regulator of RAS/ERK activation downstream of multiple receptor tyrosine kinase (RTK) and cytokine receptors [1]. Germ-line *PTPN11* mutations cause ∼50% of Noonan syndrome (NS), which is characterized by short stature, skeletal abnormalities, cardiac defects, learning disabilities, and a predisposition to hematologic abnormalities, particularly juvenile myelomonocytic leukemia (JMML) [2]. Somatic gain-of-function mutations in *PTPN11* are the most common cause of sporadic JMML in patients, and occur at lower frequency in a variety of other hematologic malignancies, including acute myelogenous leukemia and acute lymphoblastic leukemia [1], [3], [4].

Deletion of a conditional allele of *Ptpn11* in murine hematopoietic cells causes profound bone marrow (BM) aplasia, rapid loss of hematopoietic stem cells (HSC) and multi-lineage progenitors, pancytopenia and early lethality [5], [6]. Conversely, expression of leukemogenic alleles of *Ptpn11,* such as *Ptpn11^D61Y^* [7] or *Ptpn11^E76K^* [8], in the hematopoietic compartment results in a fatal myeloproliferative neoplasm (MPN), featuring leukocytosis, anemia and hepatosplenomegaly. Although anemia is a feature of global SHP2 gain-of-function expression in murine hematopoietic cells, the pleiotropic effects of mutant *Ptpn11* alleles on different hematopoietic cell lineages complicate the elucidation of their cell-autonomous roles in the erythropoiesis. To clarify the effects of oncogenic *Ptpn11* in erythroid lineage cells, we crossed the *Cre* recombinase line *ErGFPcre* to conditional *Ptpn11* gain-of-function mice.

## Materials and Methods

### Mice and cell culture

*ErGFPcre* mice [9] were crossed to LSL-*Ptpn11^D61Y^* [7] mice. Mice were backcrossed for at least 9 generations onto the C57BL/6 background and were genotyped by PCR, as described [7], [9]. All mice were maintained in accord with federal guidelines, and all animal experiments were approved by the animal welfare committee of University Health Network.

BM cells were flushed from femurs and tibiae using 26-gauge needles, and then were resuspended in 1 mL RBC lysis buffer (Sigma) for 2 minutes before PBS was added to terminate the reaction. Erythroid progenitors were cultured *in* *vitro* in StemEx medium, as described [10].

### Flow cytometry and histology

Single-cell suspensions of BM or spleen cells were prepared in PBS with 2%FBS, and stained with conjugated antibodies specific for c-KIT (2B8), SCA1 (D7), CD71 (C2) and TER119. Antibodies against lineage (Lin) markers included CD3 (145-2C11), CD4 (RM4-5), CD8α (53-6.7), CD19 (6D5), CD45/B220 (RA3-6B2) and Gr1 (Ly-6G). For intracellular flow cytometric analysis, expanded erythroblasts were starved for 1 hour, then either left unstimulated or stimulated with EPO (5 U/mL) for 5 or 15 minutes, fixed with 2% paraformaldehyde, permeabilized with 90% methanol and stained with anti-pERK, anti-pAKT, anti-pSTAT5 or anti-pSTAT3 antibodies. Flow cytometry was performed with an LSRII (Becton-Dickinson, Mountain View, CA), and data were analyzed with FlowJo software (TreeStar, Ashland, OR). Antibodies were purchased from Becton-Dickinson, eBioscience, BioLegend or Cell Signaling Technology.

Peripheral blood was analyzed with a Hemavet 950 FS hematological analyzer (Drew Scientific, Dallas, TX). The percentage of reticuloctyes in the peripheral blood was determined using Reti-COUNT Thiazole Orange Reagent (Becton-Dickinson), according the manufacturer’s instructions. The reticulocyte index was calculated as reticulocyte count (%) × (hematocrit/45). Tissues and organs were collected in 10% formalin and processed by Specialized Histopathology Services at Toronto General Hospital. Blood smears were stained with Wright-Giemsa, according to standard procedures.

### Colony assays

For CFU-E assays, 5×10^4^ BM or 1×10^5^ spleen cells were seeded in 1 mL of M3334 methyl cellulose-containing media (Stem Cell Technologies), supplemented with EPO (0.5 U). BFU-E assays were performed by seeding 5×10^4^ of BM cells in 1 mL of M3434 methyl cellulose-containing media (Stem Cell Technologies) with SCF (50 ng/mL), IL-3 (10 ng/mL) and EPO (3 U/mL). Colonies were scored after 2 (CFU-Es) or 7 (BFU-Es) days.

### Phenylhydrazine-induced stress erythropoiesis

Mice were injected intraperitoneally on day 0 and 1 with 60 mg/kg of phenylhydrazine in PBS or PBS alone. Blood and spleens were collected on days 0, 2, 4, 7, 10, 14 and 21. Hematocrit, reticulocyte count and erythroid cell frequency were determined.

### Quantitative PCR analysis

Total RNA was isolated from FACS-purified BM populations using the PicoPure Kit (Arcturus Bioscience), and subjected to reverse transcription with SuperScript III First-Strand Synthesis System (Invitrogen). qPCR assays were performed on an ABI 7500 Fast Real-Time PCR System, using the TaqMan Universal PCR master mixture (Applied Biosystem, Foster City, CA).

### Statistical analysis

Data are presented as means ± SD, and were analyzed by analysis of variance (ANOVA) with Bonferroni post-hoc test.

## Results and Discussion

SHP2 is expressed in erythroid progenitors [11], and *Ptpn11* deletion in all hematopoietic cells abrogates CFU-E formation [6]. To explore the cell-autonomous effects of a leukemia-associated *Ptpn11* mutant on the erythroid lineage, we crossed *Ptpn11^D61Y^* mice to *ErGFPcre* mice (Figure S1A); the latter express a GFP-Cre fusion protein under the control of the endogenous *Epor* promoter [9]. In *ErGFPcre*; *Ptpn11^D61Y/+^* (hereafter, *Ptpn11^D61Y^*) mice, deletion of the stop cassette in the *Ptpn11^D61Y^* allele was found in cKIT^+^CD71^+^Ter119^−^ cells (erthyroid progenitors, EP), cKIT^l^°CD71^+^ erythroblasts (ProEB), CD71^+^TER119^+^ erythroblasts (Early EB), CMP and MEP, but not in Lin^−^SCA1^+^cKIT^+^ [LSK], GMP, myeloid cells and lymphocytes (Figure S1B). Paradoxically, CD71^−^TER119^+^ cells (Late EB) cells showed inefficient expression of the mutant allele, suggesting a defect in their generation or survival (Figure S1B), with outgrowth of cells expressing the unexcised LoxP cassette. Nevertheless, *Ptpn11^D61Y^* expression was confined to the erythroid compartment, enabling us to assess its cell autonomous effects on erythropoiesis.

**Figure 1:**
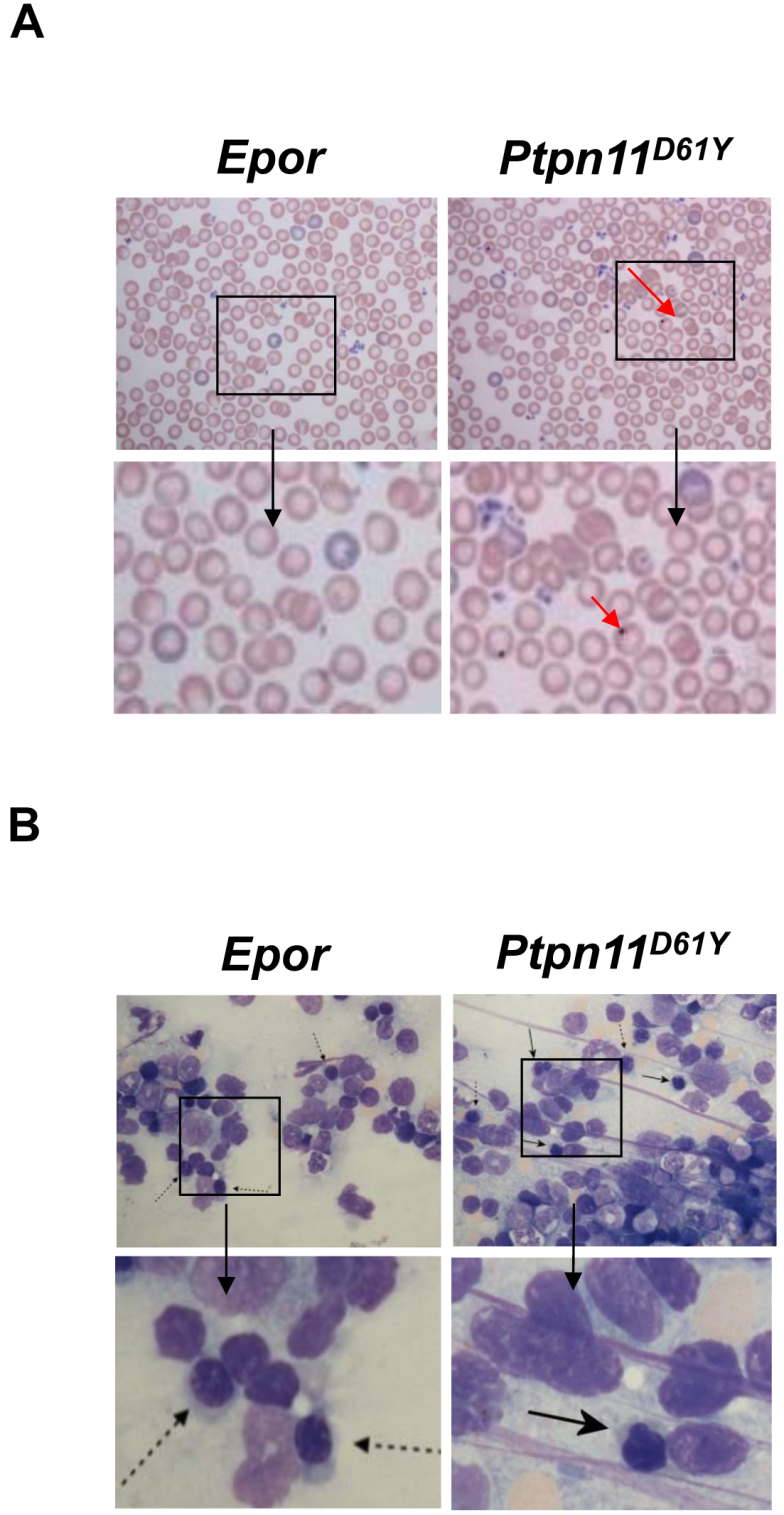
*Ptpn11^D61Y^* mice have defective erythropoiesis. (A) Howell-Jolly bodies in peripheral blood (X100) of *Ptpn11^D61Y^* mice. (B) Hematoxylin and eosin-stained BM cells (X100) from *Ptpn11^D61Y^* mice contain abnormal erythroblasts (solid arrow). Normal erythroid progenitors from control *Epor* mice are indicated (dashed arrow).

Control *ErGFPcre* (hereafter, *Epor*) mice had normal hematocrits, whereas *Ptpn11^D61Y^* mice were slightly anemic (Table 1). Peripheral blood from *Ptpn11^D61Y^* mice also showed significantly lower mean corpuscular hemoglobin (MCH) indicative of mild hypochromia (Table 1). *Ptpn11^D61Y^* RBC had Howell-Jolly bodies, and abnormal erythroblasts were present in the BM of *Ptpn11^D61Y^* mice (Figure 1A). Whereas control cells showed normal maturation with regular, round nuclei and normal chromasia, mutant BM exhibited clear dyserythropoiesis, with a large number of erythroid precursors showing nuclear abnormalities including irregular nuclear outlines, nuclear buds, binucleation, and karyorrhexis. We conclude that expression of a leukemogenic SHP2 mutant in mouse erythroid cells causes dyserythropoiesis.

**Table 1:** Hematologic parameters in peripheral blood of *Ptpn11^D61Y^* mice.

	Epor	Ptpn11D61Y
WBC (K/L)	13.4±2.5	15.0±3.1
Hb (g/dL)	13.1±2.0	11.6±2.1*
HCT (%)	43.2±4.6	34.8±6.7*
MCH (pg)	14.7±0.8	13.7±1.0*
Reticulocyte Index (%)	5.7±1.2	5.9±1.7

Peripheral blood from *Epor* (n = 18) and *Ptpn11^D61Y^* mice (n = 25) was analyzed by Hemavet 950F. Data are presented as means ± SD; (*p<0.05, by ANOVA).

**Figure 2:**
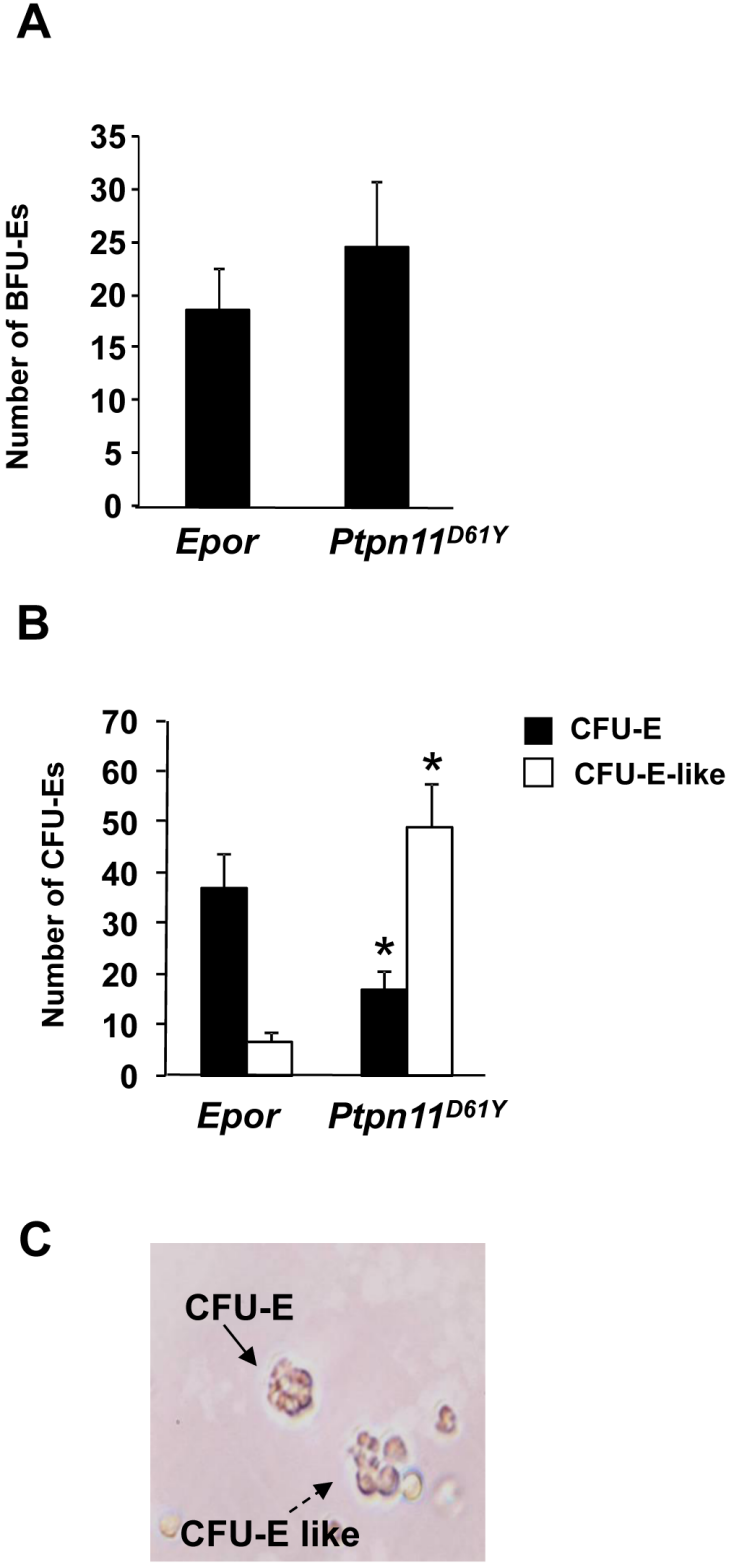
Erythroid differentiation of *Ptpn11^D61Y^* BM cells. (A) BFU-E assays performed using BM from *Epor* and *Ptpn11^D61Y^* mice. Numbers (mean ± SD, n = 6) of BFU-E per 5×10^4^ cells plated are shown. (B) CFU-E assays of BM cells from *Epor* and *Ptpn11^D61Y^* mice. Numbers (mean ± SD, n = 6) of CFU-E and CFU-like colonies per 5×10^4^ cells plated are shown. *p≤0.05, ANOVA. (C) Representative picture of CFU-E with immature phenotype.

To characterize the dyserythropoietic phenotypes further, we performed colony-forming assays. BM cells from *Epor* and *Ptpn11^D61Y^* mice produced similar numbers of BFU-Es (Figure 2A). By contrast, *Ptpn11^D61Y^* produced lower numbers of conventional CFU-Es (Figure 2B; filled columns). Instead, under standard CFU-E conditions, *Ptpn11^D61Y^*, but not *Epor*, BM cells gave rise to a large number of “CFU-E-like” colonies, which were larger and more diffuse than typical CFU-E (Figure 2C). Notably, a previous study reported that colonies with a similar morphology are associated with impaired erythropoiesis at the CFU-E stage [12].

**Figure 3:**
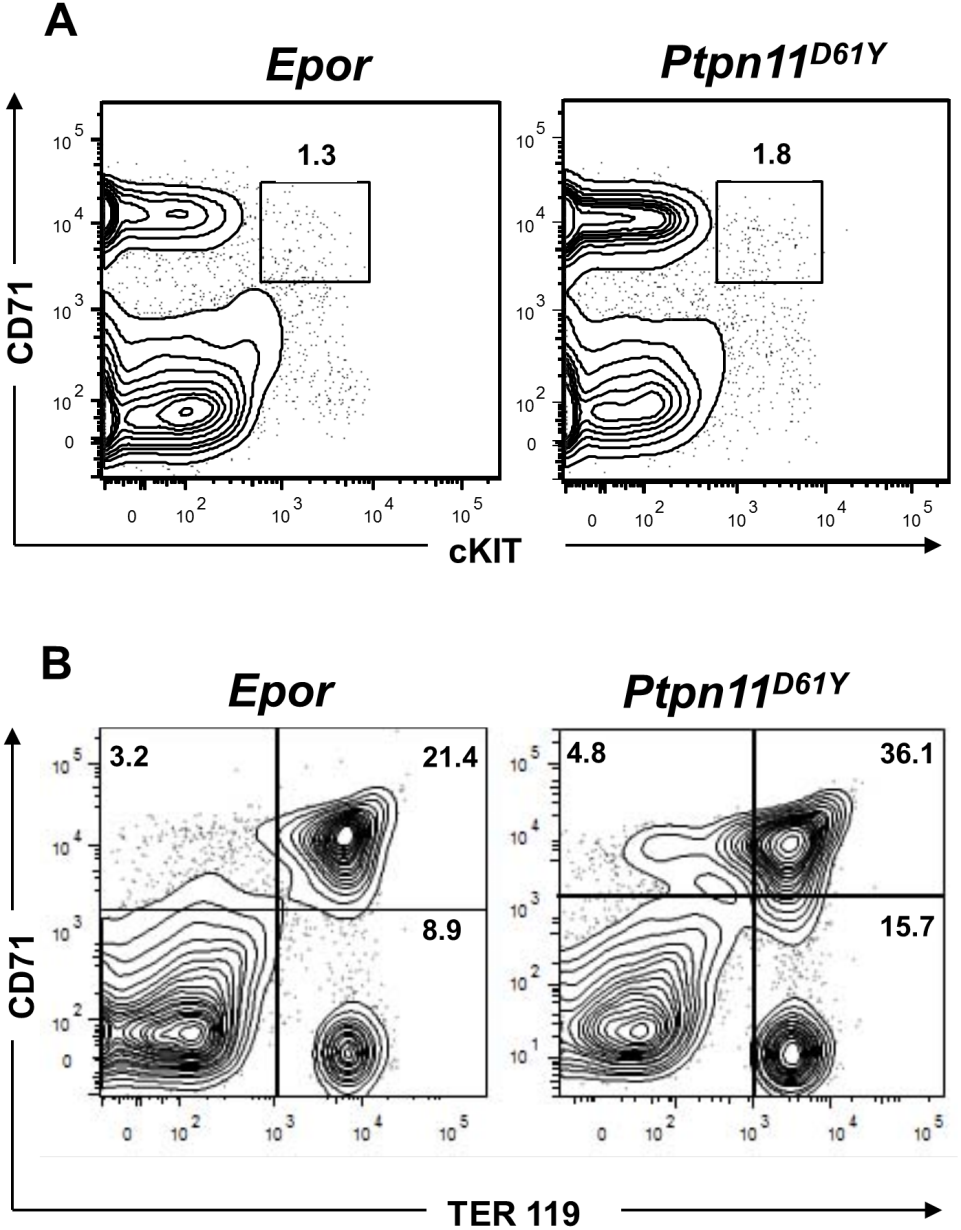
Altered erythroid differentiation of *Ptpn11^D61Y^* mice. (A, B) Representative flow cytometry profiles of BM cells from *Epor* and *Ptpn11^D61Y^* mice. Erythroid subsets include EP (cKIT^+^CD71^+^Ter119^−^) (A), and Pro EB (cKIT^l^°CD71^+^TER119^−^) and Early EB (CD71^+^TER119^+^) (B). See Table 2 for frequencies and absolute numbers of each population.

We determined the percentage and absolute numbers of erythroid cells at different stages of maturation by flow cytometry. There was no appreciable difference in the frequency and absolute number of MEPs (data not shown). Using anti-cKIT, anti-CD71 and anti-TER119 antibodies, we quantified the frequency and absolute number of more mature erythroid progenitors (Figure 3A, 3B and Table 2). There was no significant difference in total BM cellularity or in frequency or absolute numbers of Late EB in *Epor* and *Ptpn11^D61Y^* mice. However, in *Ptpn11^D61Y^* mice, the absolute numbers of EP, ProEB and Early EB were elevated compared with those in *Epor* mice (Table 2). Thus, expression of leukemogenic *Ptpn11* in the erythroid compartment results in excess production of EP, ProEB and Early EB.

**Table 2:** Frequency and absolute numbers of erythroid cells in BM of *Ptpn11^D61Y^* mutant mice.

	*Epor*	*Ptpn11^D61Y^*
Total cellularity (x10^6^)	40.4±9.9	48.5±10.2
EP (%)	1.2±0.4	1.9±0.6
EP (x10^6^)	0.5±0.2	0.9±0.4*
ProEB (%)	3.2±2.6	4.2±1.4*
ProEB (x10^6^)	1.3±0.6	2.0±0.7*
Early EB (%)	25.9±9.6	34.2±7.7*
Early EB (x10^6^)	10.5±3.8	16.6±3.7*
Late EB (%)	17.3±9.0	21.5±9.6
Late EB (x10^6^)	6.6±3.3	10.7±4.7

Frequencies and absolute numbers of BM erythroid cells from *Epor* (n = 6) and *Ptpn11^D61Y^* mice (n = 9) mice. Data are presented as means ± SD (*p<0.05, by ANOVA).

We also tested the effect of these mutations on stress erythropoiesis. Hemolysis was induced by injecting *Epor* and *Ptpn11^D61Y^* mice with phenylhydrazine (PHZ). At the indicated times, peripheral blood and spleens were collected, and HCT and the percentage of CD71^+^ and Ter119^+^ spleen cells were determined. Both groups of mice showed a similar decrease in HCT at 2 days post-PHZ treatment (Figure S2A). Likewise, by day 6, HCT had recovered to pre-treatment levels in all mice, regardless of genotype. Notably, *Ptpn11^D61Y^* mice showed significantly enhanced CD71^+^ cells at Day 2 compared with control mice, consistent with enhanced sensitivity to erythrogenic stimuli. By Day 4, though, the frequency of CD71^+^ cells in control and mutant mice was comparable (Figure S2B). *Epor* and *Ptpn11^D61Y^* mice showed similar PHZ-induced increases in the frequency of Ter119^+^ cells, which returned to basal levels by day 14 (Figure S2C). Thus, the expression of the *Ptpn11^D61Y^* allele perturbs normal erythropoiesis and has mild stimulatory effects on stress erythropoiesis.

**Figure 4:**
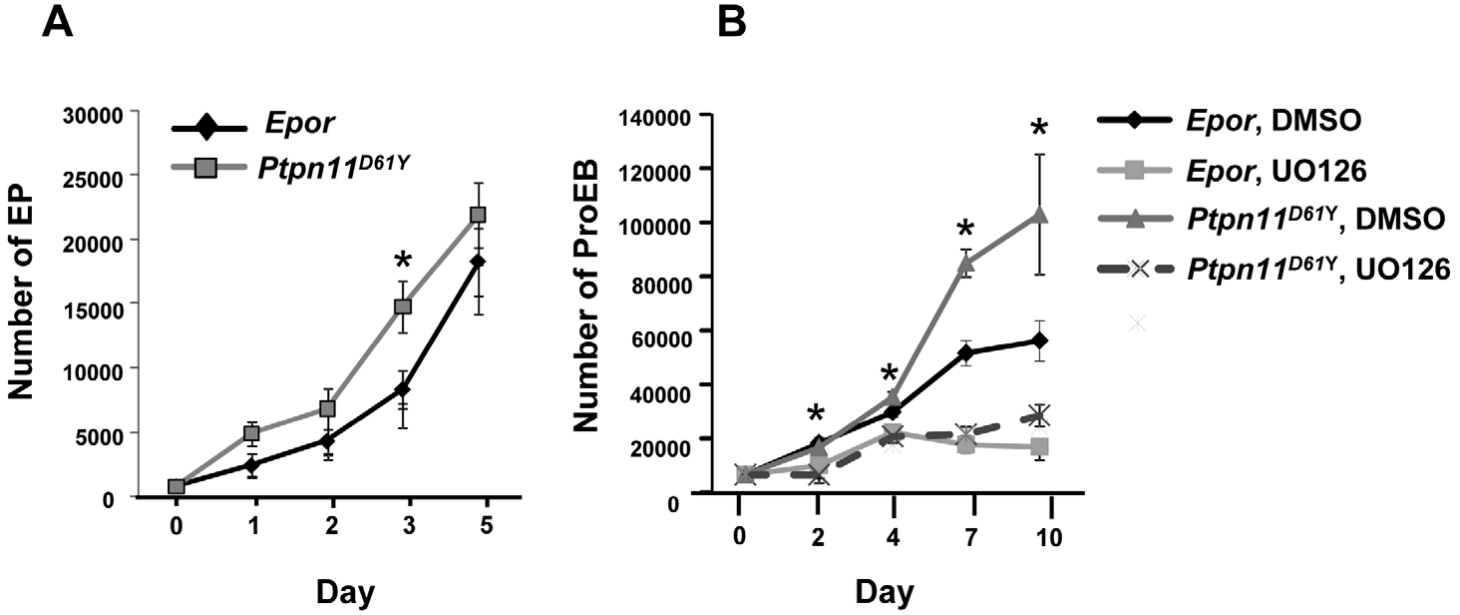
*Ptpn11^D61Y^* erythroid progenitors show defective differentiation *in* ***vitro*** **.** (A) BM-derived Lin^−^cKIT^+^ cells from *Epor* and *Ptpn11^D61Y^* mice were FACS-purified and cultured in serum-free StemEx medium for 5 days, as described in Materials and Methods. Absolute numbers of cells were determined daily. The average number of cells from four independent experiments is shown. *p≤0.05, ANOVA. (B) Lin^−^cKIT^+^ cells from *Epor* and *Ptpn11^D61Y^* mice were FACS-purified and cultured in serum-free StemEx medium 7 days. Equal numbers of cells were expanded in StemEx medium for another 10 days in the presence of UO126 or DMSO. The average number of cells from three independent experiments is shown. *p≤0.05, ANOVA.

To further assess the mechanism underlying defective erythropoiesis in *Ptpn11* mutant mice, we purified lin^−^cKIT^+^ BM cells by FACS and cultured them in StemEx medium (Figure 4A). Under these conditions, phenotypic EP and ProEB are generated in a stepwise fashion from lin^−^cKIT^+^ progenitors over 3–5 days [13]. Compared with *Epor* cells, lin^−^cKIT^+^ cells from *Ptpn11^D61Y^* mice produced significantly higher numbers of phenotypic EP after three days of culture (Figure 4A), suggesting that the increased numbers of EP in *Ptpn11^D61Y^* mice (Figure 1C) reflect excess production of these cells from an earlier progenitor.

Next, we assessed the proliferative potential of cells harvested after seven days of StemEx culture, at which time >90% have the phenotype of ProEB (data not shown). These cells were allowed to proliferate for another 10 days (Figure 4B). Compared with *Epor* cells, the *Ptpn11^D61Y^* ProEB showed markedly increased proliferation. Thus, similar to what is observed *in* *vivo*, the expansion of ProEB *in* *vitro* is enhanced by the expression of *Ptpn11^D61Y^*. Studies of Mx1-Cre; *Kras^G12D^* [14], [15] mice also revealed enhanced levels of ProEB, although those studies could not determine whether such effects are erythroid lineage-specific. Nevertheless, the similarities between the erythroid phenotype in *Kras^G12D^* and *Ptpn11^D61Y^* mice are consistent with the known critical role for SHP2 in regulating the RAS/ERK pathway.

**Figure 5:**
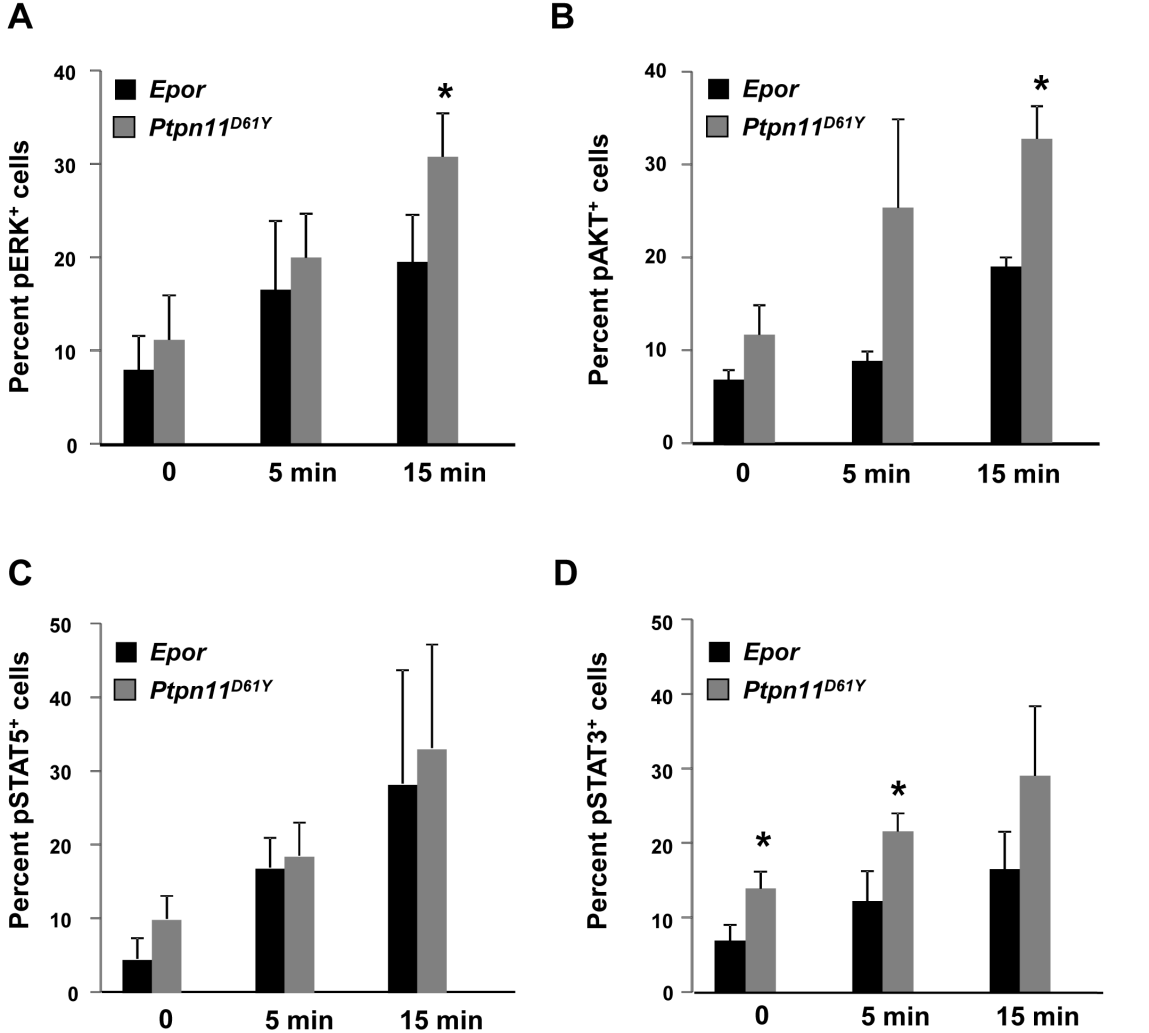
Signaling aberrations in *Ptpn11^D61Y^* erythroid progenitors. (A–D) CD71^+^ cells generated in *ex* *vivo* cultures were starved for 6 hours were either left untreated or stimulated with EPO (2.5 U/mL). Cells were fixed, permeabilized and stained with anti-pERK (A), -pAKT (B), -pSTAT5 (C), and -pSTAT3 (D) antibodies. Levels of phospho-specific antigens in these cells were determined by flow cytometry. The average percentages of phospho-specific signals from four experiments are shown as mean ± SD, *p<0.05, ANOVA.

Next, we interrogated signalling pathways downstream of the EPOR using multi-parameter flow cytometry (Figure 5A–D). EPO-evoked ERK and AKT activation were enhanced in *Ptpn11^D61Y^* cKIT^l^°CD71^+^ progenitors (Figure 5A, B). Although these cells showed normal levels of EPO-induced STAT5 phosphorylation (Figure 5C), EPO-evoked STAT3 activation was elevated in *Ptpn11^D61Y^* cells (Figure 5D). Hence, *Ptpn11* mutant erythroid progenitors show perturbed EPOR signaling, which is consistent with their effect on erythropoiesis *in* *vitro* and *in* *vivo*.

To determine whether reducing ERK activation in *Ptpn11^D61Y^* cells could normalize their proliferative responses, erythroid progenitors were allowed to expand in the presence of vehicle or the MEK inhibitor UO126 (Figure 4B). Compared with DMSO-treated cells, UO126-treated *Ptpn11^D61Y^* cells showed markedly reduced proliferation (Figure 4B). Thus, excessive EPO-evoked ERK activation is critical for the enhanced proliferation of *Ptpn11^D61Y^* erythroid progenitors.

In summary, expression of *Ptpn11^D61Y^* leads to enhanced EPO-evoked ERK, AKT, and STAT3 activation and excessive production of EP, ProEB and Early EB, resulting in altered erythropoiesis *in* *vivo*. Our results show that all of these effects reflect the action of mutant SHP2 directly on erythroid lineage cells, and, together with previous results [7], [8], argue that JMML arises from the combined effects of leukemogenic SHP2 in multiple lineages and stages of hematopoiesis.

## Supporting Information

Figure S1 **Generation of** ***Ptpn11^D61Y^*** **mice**. (A) Schematic of breeding for the generation of *_Ptpn11_^D61Y^* mice. (B) Assessment of STOP-cassette deletion in *Ptpn11^D61Y^* mice. Hematopoietic cells were purified by FACS and DNA was extracted. Deletion of the STOP cassette was assessed by PCR (“C”, *Epor*, “D”, *Ptpn11^D61Y^*).
(PDF)

Figure S2 **Stress erythropoiesis in** ***Ptpn11^D61Y^*** **mutant mice.** (A) *Epor* (n = 7) and *Ptpn11^D61Y^* (n = 5) mice were treated with PHZ for the indicated times and the kinetics of hematocrit changes were determined. (B–C) The frequency of CD71^+^ (B) and Ter119^+^ (C) in the spleens of *Epor* (n = 4) and *Ptpn11^D61Y^* (n = 3) mice were determined at the indicated times following PHZ treatment. Data are presented as mean±SD, and were analyzed by analysis of variance (ANOVA) with Bonferroni post-hoc test; *p≤0.05.
(PDF)
