# Supplementary material for: Leukemogenic Ptpn11 Allele Causes Defective Erythropoiesis in Mice
Source: PLoS One. 2014 Oct 7;9(10):e109682. doi: 10.1371/journal.pone.0109682 (PMC4188809; doi:10.1371/journal.pone.0109682)
Supplement: Figure S1 — Generation of Ptpn11D61Y mice. (A) Schematic of breeding for the generation of Ptpn11D61Y mice. (B) Assessment of STOP-cassette deletion in Ptpn11D61Y mice. Hematopoietic cells were purified by FACS and DNA was extracted. Deletion of the STOP cassette was assessed by PCR (“C”, Epor, “D”, Ptpn11D61Y). (PDF) [file pone.0109682.s001.pdf]

Figure S1

A

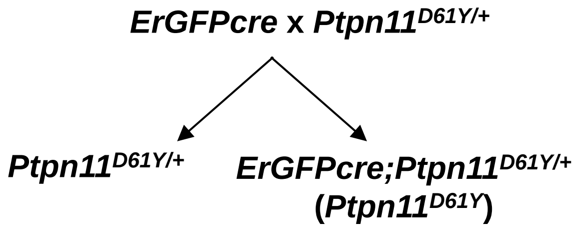

B

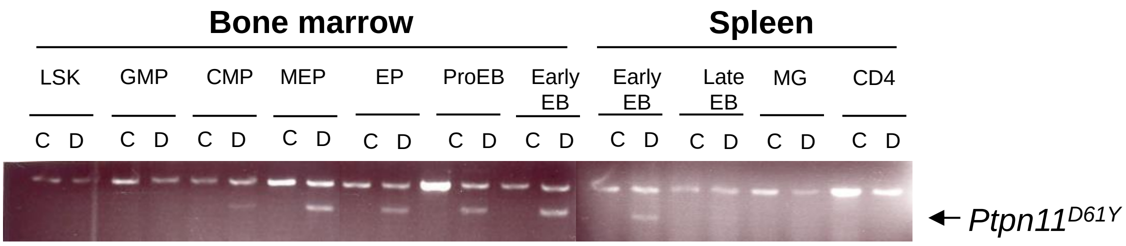

Figure S1. Generation of *Ptpn11^{D61Y}* mice.

(A) Schematic of breeding for the generation of *Ptpn11^{D61Y}* mice.

(B) Assessment of STOP-cassette deletion in *Ptpn11^{D61Y}* mice. Hematopoietic cells were purified by FACS and DNA was extracted. Deletion of the STOP cassette was assessed by PCR (“C”, *Epor*, “D”, *Ptpn11^{D61Y}*).
