# Supplementary material for: Leukemogenic Ptpn11 Allele Causes Defective Erythropoiesis in Mice
Source: PLoS One. 2014 Oct 7;9(10):e109682. doi: 10.1371/journal.pone.0109682 (PMC4188809; doi:10.1371/journal.pone.0109682)
Supplement: Figure S2 — Stress erythropoiesis in Ptpn11D61Y mutant mice. (A) Epor (n = 7) and Ptpn11D61Y (n = 5) mice were treated with PHZ for the indicated times and the kinetics of hematocrit changes were determined. (B–C) The frequency of CD71+ (B) and Ter119+ (C) in the spleens of Epor (n = 4) and Ptpn11D61Y (n = 3) mice were determined at the indicated times following PHZ treatment. Data are presented as mean±SD, and were analyzed by analysis of variance (ANOVA) with Bonferroni post-hoc test; *p≤0.05. (PDF) [file pone.0109682.s002.pdf]

Figure S2

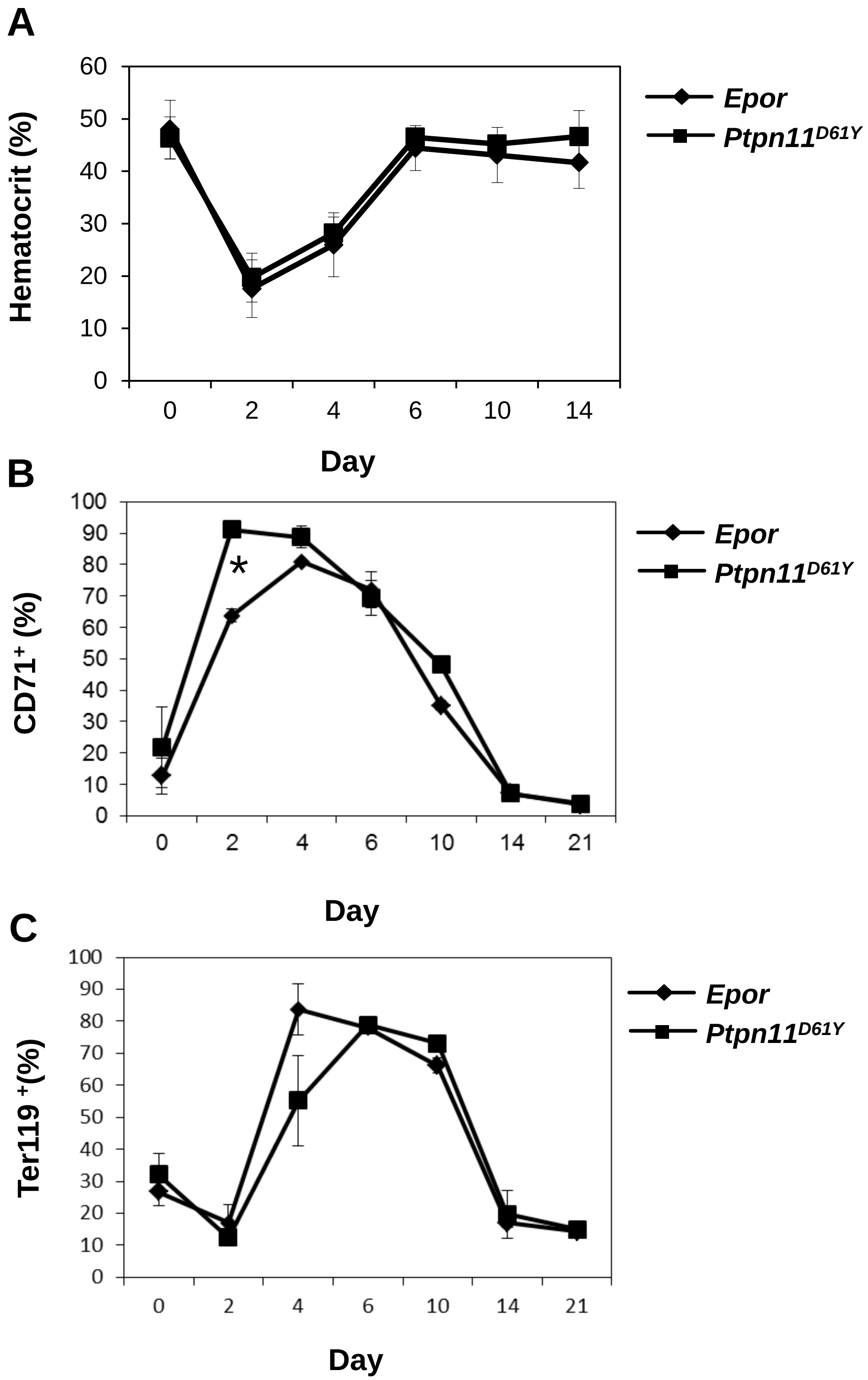

Figure S2. Stress erythropoiesis in *Ptpn11^{D61Y}* mutant mice.

(A) *Epor* (n=7) and *Ptpn11^{D61Y}* (n=5) mice were treated with PHZ for the indicated times and the kinetics of hematocrit changes were determined. (B – C) The frequency of CD71^{+} (B) and Ter119^{+} (C) in the spleens of *Epor* (n=4) and *Ptpn11^{D61Y}* (n=3) mice were determined at the indicated times following PHZ treatment. Data are presented as mean±SD, and were analyzed by analysis of variance (ANOVA) with Bonferroni post-hoc test; *$p \leq 0.05$.
